# Ameliorative Effects of Camel Milk and Its Exosomes on Diabetic Nephropathy in Rats

**DOI:** 10.3390/membranes12111060

**Published:** 2022-10-28

**Authors:** Amira M. Shaban, Mai Raslan, Safa H. Qahl, Khaled Elsayed, Mohamed Sayed Abdelhameed, Atif Abdulwahab A. Oyouni, Osama M. Al-Amer, Ola Hammouda, Mohammed A. El-Magd

**Affiliations:** 1 Biotechnology & Life Sciences Department, Faculty of Postgraduate Studies for Advanced Sciences, Beni-Suef University, Beni-Suef 62511, Egypt; 2 Department of Biology, College of Science, University of Jeddah, Jeddah 21589, Saudi Arabia; 3 Botany and Microbiology Department, Faculty of Science, Beni-Suef University, Beni-Suef 62511, Egypt; 4 Department of Biology, Faculty of Sciences, University of Tabuk, Tabuk 47512, Saudi Arabia; 5 Genome and Biotechnology Unit, Faculty of Sciences, University of Tabuk, Tabuk 47512, Saudi Arabia; 6 Department of Medical Laboratory Technology, Faculty of Applied Medical Sciences, University of Tabuk, Tabuk 47512, Saudi Arabia; 7 Anatomy Department, Faculty of Veterinary Medicine, Kafrelsheikh University, El-Geish Street, Kafrelsheikh 33516, Egypt

**Keywords:** camel milk, exosomes, diabetic nephropathy, oxidative stress, fibrosis

## Abstract

Contradictory results were obtained regarding the effects of extracellular vesicles such as exosomes (EXOs) on diabetes and diabetic nephropathy (DN). Some studies showed that EXOs, including milk EXOs, were involved in the pathogenesis of DN, whereas other studies revealed ameliorative effects. Compared to other animals, camel milk had unique components that lower blood glucose levels. However, little is known regarding the effect of camel milk and its EXOs on DN. Thus, the present study was conducted to evaluate this effect on a rat model of DN induced by streptozotocin. Treatment with camel milk and/or its EXOs ameliorated DN as evidenced by (1) reduced levels of kidney function parameters (urea, creatinine, retinol-binding protein (RBP), and urinary proteins), (2) restored redox balance (decreased lipid peroxide malondialdehyde (MDA) and increased the activity of antioxidants enzymes superoxide dismutase (SOD), catalase (CAT) and glutathione peroxidase (GPx)), (3) downregulated expression of DN-related genes (transforming growth factor-beta 1 (*TGFβ1*), intercellular adhesion molecules 1 (*ICAM1*), and transformation specific 1 (*ETS1*), integrin subunit beta 2 (*ITGβ2*), tissue inhibitors of matrix metalloproteinase 2 (*TIMP2*), and kidney injury molecule-1 (*KIM1*)), and (4) decreased renal damage histological score. These results concluded that the treatment with camel milk and/or its EXOs could ameliorate DN with a better effect for the combined therapy.

## 1. Introduction

Persistent hyperglycemia in diabetic patients induces diabetic complications that negatively affect health outcomes [1]. Diabetic nephropathy (DN) is one of the most common microvascular problems in diabetic patients and is the main cause of end-stage kidney diseases and renal failure [2]. The key features of DN are prolonged proteinuria and glomerulosclerosis (thickened glomerular basement membrane). The main pathological lesions of DN involve abnormal mesangial cell proliferation, which initiates extracellular matrix aggregation and destroys podocytes, leading to glomerular sclerosis and tubulointerstitial fibrosis [3]. There is a close association between fibrosis/matrix remodeling and the progression of DN. Fibrosis-related genes (such as *TGFβ1* and *ICAM1*), matrix remodeling-related genes (such as *ETS1*, *ITGβ2*, and *TIMP2*), and the kidney injury-related gene (*KIM1*) play important roles in DN pathogenesis [4,5,6]. Many treatment strategies targeting cytokines, chemokines, and cell adhesion molecules revealed beneficial results in experimental models of DN, lowering proteinuria and glomerulosclerosis [7]. Traditional therapy targeting hypertension and hyperglycemia do not prevent or reverse DN.

Consumption of animal milk has beneficial effects on the health of diabetic patients. Brown fat was raised, glucose and fructosamine levels were lowered, and glucose tolerance was enhanced in diabetic animals that consumed cow milk [8]. Cow fermented milk is rich in bioactive peptides that can also lower glucose level and could prevent or treat type 2 diabetes [9]. Similar to cow milk, previous studies also reported an effective role of camel milk (CM) in the control of diabetes in humans and animals [10,11,12]. Insulin-dependent diabetic patients who regularly consumed CM required 30–35% insulin less than other diabetic patients who did not consume CM [11,13]. Additionally, streptozotocin (STZ)-induced diabetic rats consuming CM for 30 days possessed lower blood glucose levels and higher insulin sensitivity [11,14,15,16]. CM hypoglycemic effect was attributed to the presence of insulin and insulin-like substances and the lack of CM coagulation and degradation in the stomach [17,18]. Micelles may shield CM insulin from stomach digestion and aid intestinal absorption [18,19]. Some of the hypoglycemic effects of CM may also result from its potent antioxidant, anti-inflammatory, and hypolipidemic properties [20].

Extracellular vesicles (EVs) are lipid-bound vesicles (40–150 nm) secreted by nearly all types of cells into the extracellular space mainly in the form of microvesicles and exosomes (EXOs) [21,22,23]. EXOs play essential roles in cell-to-cell communication [24,25,26,27]. Their phospholipid layer protects their nucleic acid cargo, notably miRNAs, from gastrointestinal breakdown and aids intestine absorption [28,29]. Milk EXOs can influence the milk recipient’s immune system through their cargo of immune-regulatory miRNAs and can effectively alleviate the inflammatory response [30,31]. Furthermore, the antioxidant activity and most biochemical and immunological parameters in the cyclophosphamide-injected rats were restored by the camel milk EXOs [32]. Aside from their immunomodulatory effects, the role played by EXOs in renal damage diseases, including DN, showed inconsistent results. Previous studies reported positive roles for EXOs derived from both pancreatic β-cells and stem cells in the pathogenesis of diabetes [33,34] and DN [35,36,37,38]. In contrast, other studies reported ameliorative effects for EXOs derived from bone marrow mesenchymal stem cells (BM-MSCs) on DN and renal ischemia-reperfusion injury in rats [39,40]. MSCs-derived EXOs in rats induced MAPK signaling pathway and β-cell propagation [41]. Additionally, camel milk EXOs also induce the proliferation of normal human pancreatic H6c7 cells [42] and cow EXOs were recently used to successfully deliver insulin orally to diabetic rats [43].

A previous study reported that CM could ameliorate renal damage induced by STZ in a rat model of DN [44]. As previously mentioned, CM had unique constituents (such as insulin) and health-promoting properties (antioxidant and anti-inflammatory) which could explain its hypoglycemic potential and ameliorative effects against DN. However, the effect of CM-derived EXOs on DN and the possible underlying molecular mechanisms have not been addressed yet. Thus, the present study was conducted to evaluate the effect of CM and its EXOs on STZ-induced DN in rats.

## 2. Materials and Methods

### 2.1. Isolation of Milk-Derived Exosomes

Fresh milk samples were collected from normally lactating she-camels (*Camelus dromedaries*) at the mid-lactation period from a national local camel farm in Marsa Mattrouh, Egypt. EXOs were isolated from milk by ultracentrifugation as previously described [45,46]. In brief, the milk samples were first centrifuged at 5000× *g* for 15 min, then at 13,000× *g* for 30 min at 4 °C to take away somatic cells, casein, and other debris. EXOs had been isolated from supernatants via two ultracentrifugations at 100,000× *g* (Optima L-90okay; Beckman Coulter) at 4 °C for 90 min each with an interval step of phosphate-buffered saline (PBS) wash to remove fat globules, and larger milk vesicles. The EXO pellets were resuspended in PBS. Exosomal protein concentration was determined by a BCA kit and aliquots of EXOs at a concentration less than 6 mg/mL were kept at −80 °C until use.

### 2.2. Characterization of Milk-Derived Exosomes

The diameter and shape of the EXOs were determined by using transmission electron microscopy (TEM). Before the examination, EXOs were fixed in 2.5% glutaraldehyde/2 h/room temperature, then 10 μL EXOs were mounted on a carbon-coated copper grid covered with nitrocellulose and examined by TEM (JEM2100, Joel Inc., Japan) at 80 kV. Size distribution of EXOs was measured by dynamic light scattering (DLS) by using a Nano Zeta Sizer System (Malvern Instruments) with a 633-nm laser wavelength and a 173° scattering angle. EXOs were further characterized by the detection of specific exosomal proteins CD63 and CD81 by using flow cytometry. In brief, EXOs were incubated with either anti-CD63 (1:200, Santa Cruz, Germany) or anti-CD81 (1:200, Santa Cruz) for 1 h in the dark at room temperature. Data were detected by an Attune flow cytometer (Applied Biosystem, San Francisco, CA, USA), and a standard nanobead calibration kit containing beads (50 and 100 nm, Technologies Drive Fisher, Lenexa, KS, USA) was used to set the gate and count EXOs.

### 2.3. Chemical Induction of Diabetic Nephropathy

Rats were intraperitoneally (IP) injected with a single dose of STZ at a dose of 60 mg/kg body weight (BW) dissolved in 0.1 mol/L cold citrate buffer, pH 4.5 (Sigma-Aldrich Chemical Co). The volume of STZ injected was 0.25 mL/kg BW. Sucrose (15 g/L) was given to rats in drinking water for 2 days to decrease the death of rats from pancreatic β-cell damage induced by STZ. After 3 days of STZ injection, rats with fasting blood glucose of more than 250 mg/dL were considered diabetic and involved in the experiment. The desirable level of blood glucose (350 mg/dL) was maintained by subcutaneous injection of diabetic rats with long-lasting insulin (3 U/rat) to avoid ketonuria [47].

### 2.4. Experimental Design

Experimental protocols were approved by the Ethical Committee at the Faculty of Veterinary Medicine, Kafrelsheikh University, Egypt with a license number of 32971/273. A total number of 60 male albino rats were enrolled in this study. Animals were kept in optimal environmental conditions (25 °C ± 2, 12-h light/dark cycle) and received a basal diet and water ad libitum. They were acclimatized to laboratory conditions for 2 weeks before the experiment. Rats were randomly divided into 5 groups. In the control (Cnt) group (n = 15), rats were IP injected with a single dose of 0.25 mL/kg body weight (BW) sodium citrate buffer as a vehicle. In the DN group (n = 15), rats were IP injected with a single dose of STZ (0.25 mL/kg BW) and left for 12 weeks without treatment. At the seventh week, 5 rats from this group and 5 from the Cnt group were euthanized by exsanguination and blood samples were collected and kidneys were dissected to confirm the occurrence of DN as revealed by significant increases in proteinuria and glomerular sclerosis relative to the Cnt group. In the CM group (n = 10), rats were injected with STZ as in the DN group and at the eighth week, they were orally administered CM (10 mL/rat/day) for one month, until the twelfth week [44]. In the EXO group (n = 10), rats were injected with STZ as in the DN group and at the eighth week, they were orally administered 1 mL camel milk EXOs (1.25 mg/kg BW) once per week for 1 month, until the twelfth week [32]. In the CM + EXO group (n = 10), rats were injected with STZ as in the DN group and at the eighth week, they were orally administered CM and EXOs as previously mentioned.

### 2.5. Sampling

At the end of the twelfth week, urine samples were collected from rats placed in metabolic cages. Blood samples were collected from the medial canthus of the eyes by capillary tubes and some samples were centrifuged at 3000 rpm for 5 min to get serum for the detection of kidney function parameters, and other whole blood samples were used to estimate fasting blood glucose. Rats were euthanized by exsanguination and kidneys were quickly excised, and some renal tissue specimens were homogenized, centrifuged at 12,000× *g* for 15 min at 4 °C, and the supernatant was used for the biochemical assay. Other kidney specimens were snap-frozen in liquid nitrogen for real time PCR or preserved in 10% formalin for histological examination.

### 2.6. Biochemical Parameters

The level of fasting blood glucose was determined by using a commercially available kit (Biodiagnostic, Egypt). Serum levels of creatinine and urea were estimated calorimetrically by using commercially available kits (Bio-Med, Egypt). Retinol-binding protein (RBP) concentration in serum was measured by an ELISA kit (LSBio, Inc., Seattle, WA, USA, # LS-F28065) according to the standard protocol of the manufacturer. The concentration of proteins in the urine was estimated based on the manufacturer’s instructions (Fortress Diagnostics Ltd., Antrim, UK). The levels of lipid peroxidation biomarker malondialdehyde (MDA) and the activity of the antioxidant enzymes SOD, CAT, and GPx were measured in kidney homogenates by using commercially available kits (Biodiagnostics, Egypt) as previously detailed [48,49].

### 2.7. Real-Time PCR

The renal expression of fibrosis-related genes (*TGFβ1* and *ICAM1*), matrix remodeling genes (*ETS1*, *ITGβ2*, and *TIMP2*), and the kidney injury-related gene (*KIM1*) following treatment with CM and/or its EXOs were detected by real-time PCR. First, total RNA was extracted from kidney specimens by using a Trizol reagent (Invitrogen, USA, Cat# 15596026). RNA concentration and purity were determined by using a Nanodrop (Q5000, Quawell, USA). Secondly, cDNA was synthesized from RNA by using the RevertAid H Minus Reverse (Thermo Scientific, #EP04 51). Finally, a PCR mix containing cDNA, 2XMaster Mix (QuantiTect SYBR Green), and primers (Table 1) was run in the Step One Plus thermal cycler (Applied Biosystem, USA) at the following conditions: one cycle initial denaturation at 94 °C for 4 min followed by 40 cycles of each denaturation at 94 °C for 40 s, annealing at 60 °C for 30 s, and extension at 72 °C for 30 s. The housekeeping gene *β actin* was utilized as an internal control. The results of qPCR were presented as fold change mean ± standard error of the mean (SEM) by using the Livak method (2^−∆∆Ct^) and as previously described [50].

### 2.8. Histopathology

Paraffin sections (5 µm) were prepared from overnight 10% formalin fixed kidney specimens. All tissue slides were stained with hematoxylin and eosin and blindly examined by a light microscope. The renal damage score involved the following lesions (1) mononuclear cells infiltration, (2) glomerular sclerosis, (3) mesangial cells hyperplasia, (4) cell desquamation in renal tubules, and (5) cast in renal tubules. These histopathological changes were determined in 10 randomly chosen non-overlapping fields which were examined at 40×. The damage score was set as follows: 0 (no damage), 1 (≤10% damage), 2 (11–25% damage), 3 (26–45% damage), 4 (46–75% damage), and 5 (≥76% damage) [51].

### 2.9. Statistical Analysis

Data were checked for normal distribution before performing statistical analysis. One-way ANOVA followed by Tukey’s honestly significant difference as a post-doc test was applied by using GraphPad Prism 8 software to determine significant differences among the experimental groups. Data were presented as mean per group ± standard error of the mean (SEM), and the significance was declared at *p* < 0.05.

## 3. Results

### 3.1. Characterization of CM-Derived EXOs

**Figure 1:**
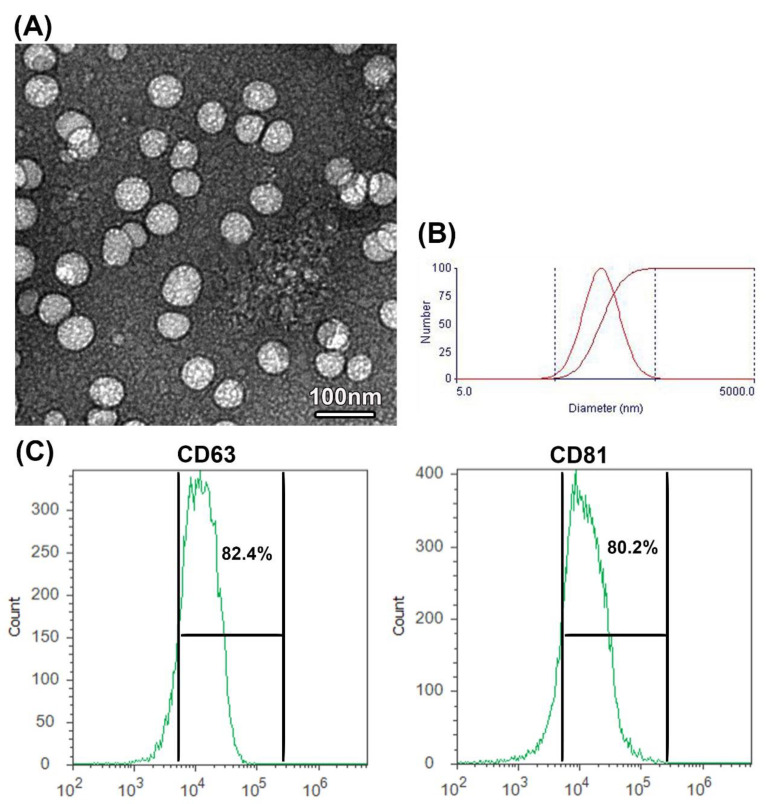
Characterization of EXOs isolated from camel milk. (**A**) TEM photograph shows camel milk-derived EXOS isolated by differential ultracentrifugation, scale bar = 100 nm. (**B**) DLS photograph shows camel milk EXOs with various sizes ranging from 35 to 130 nm. The first curve indicates the differential distribution, and the second curve indicates the cumulative distribution (**C**) Detection of specific exosome proteins by flow cytometry showing percentage of positive exosomal CD63 and CD81 proteins.

As examined under TEM, camel milk EXOs appeared as nanospheres with various sizes ranging from 50 to 100 nm (Figure 1A). Size distribution, as detected by DLS, showed EXOs with different sizes ranging from 35 to 130 nm with a mean diameter of 87.3 ± 7.20 nm (Figure 1B). The flow cytometry analyses (Figure 1C) exhibited the presence of a high percentage of specific exosomal marker proteins CD63 (82.4%) and CD81 (80.2%). These results implied the effective isolation of EXOs from the camel milk.

### 3.2. Effects of CM and/or Its EXOs on Kidney Damage Parameters

Levels of kidney damage parameters (urea, creatinine, retinol-binding protein (RBP), and urinary proteins) and fasting blood glucose were significantly higher in STZ-injected rats (DN group) than in the control (Cnt) group (Table 2). The levels of these parameters were significantly decreased in the three treated groups, with lowest levels in the co-treated (CM + EXO) group, compared to the DN group. The EXO group also showed significantly decreased urea and RBP levels relative to the CM group. However, the levels of all parameters in the treated groups remained higher than in the control group. These results inferred that treatment with CM and/or its EXOs attenuated the elevated levels of kidney damage parameters and blood glucose induced by STZ in DN rats.

### 3.3. Effects of CM and/or Its EXOs on Oxidative Stress and Antioxidants Parameters

**Figure 2:**
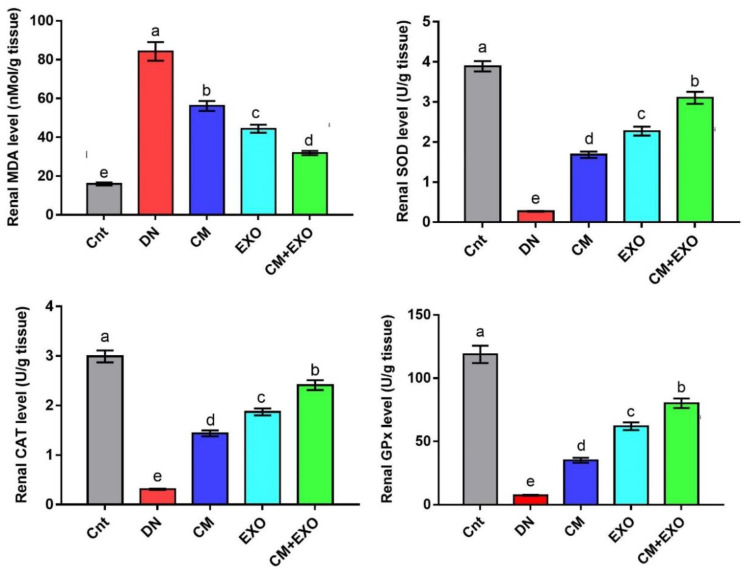
Effect of camel milk and/or its EXOs on oxidative stress (MDA) and antioxidant (SOD, CAT, and GPx) status in rat renal tissue. Values were expressed as mean ± SEM (n = 6/group). Columns carrying different letters (a (the highest value)–e (the lowest value)) are significantly different at *p* < 0.05. All groups were compared to each other. Each data point represents an average of three independent experiments with three replicates for each. Cnt, control group; DN, diabetic nephropathy group; CM, camel milk-treated group; EXO, camel milk exosomes-treated group; CM + EXO, co-treated group with camel milk and its exosomes.

Rats in the DN group had significantly higher renal levels of the lipid peroxidation biomarker MDA and significantly lower renal levels of the antioxidant enzymes (SOD, CAT, and GPx) than the control group (Figure 2). Treatments with CM and its EXOs each alone or in combination (CM and EXO) partially normalized the redox balance as indicated by the reduction of MDA and elevation of the antioxidant enzymes relative to the DN group. The co-treated group (CM and EXO) significantly achieved the best improvement followed by the EXO group compared to the CM group. However, none of the treatments returned the levels of these oxidative and antioxidant markers to normal levels. These results implied that CM and/or its EXOs restored oxidative stress associated with DN with the best effect when given together.

### 3.4. Effect of CM and/or Its EXOs on Fibrosis and Kidney Damage-Related Genes

**Figure 3:**
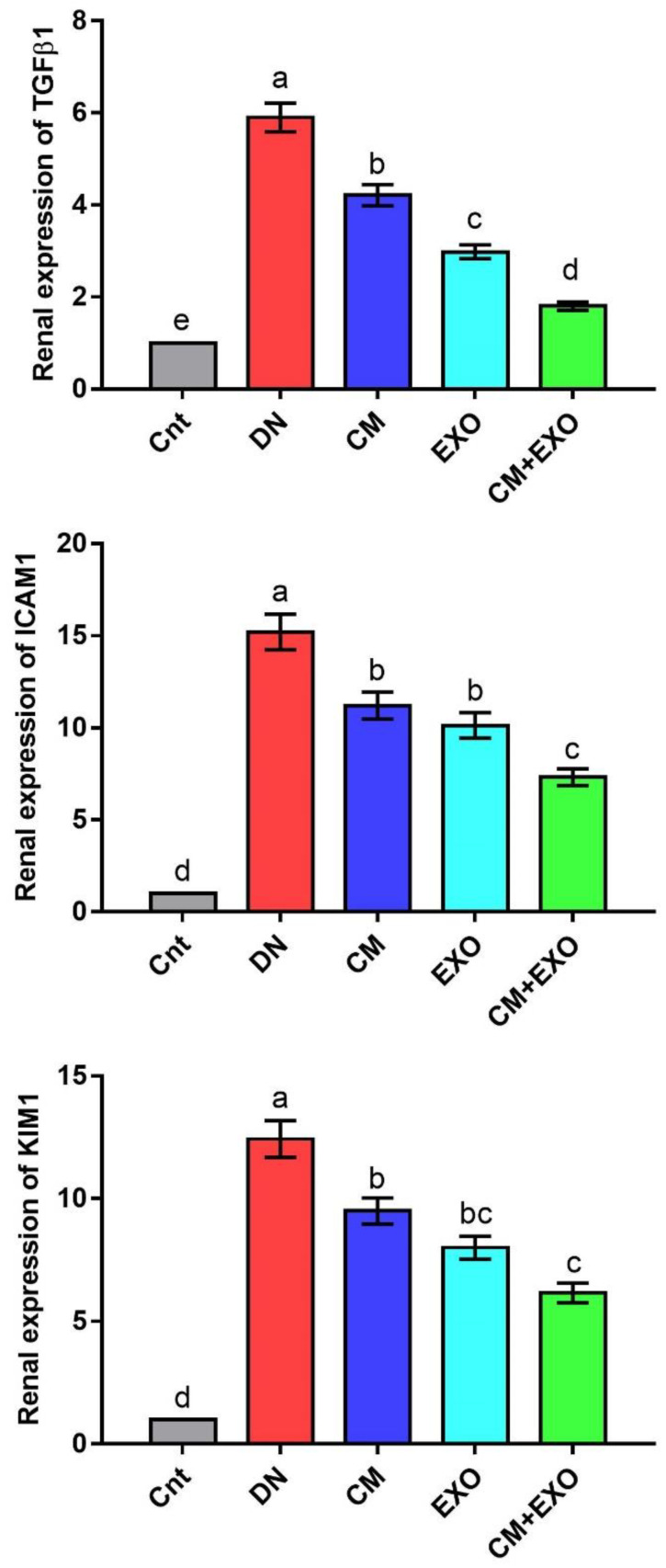
Effect of camel milk and/or its EXOs on renal expression of fibrosis-related genes (*TGFβ1* and *ICAM1*) and the kidney injury-related gene (*KIM1*) as detected by real-time PCR. Values were expressed as fold change mean ± SEM (n = 6/group). Columns carrying different letters (a (the highest value)–e (the lowest value)) are significantly different at *p* < 0.05. All groups were compared to each other. Each data point represents an average of three independent experiments with three replicates for each. Cnt, control group; DN, diabetic nephropathy group; CM, camel milk-treated group; EXO, camel milk exosomes-treated group; CM + EXO, co-treated group with camel milk and its exosomes.

The changes in relative renal expression of the fibrosis-related genes (*TGFβ1* and *ICAM1*) and the kidney injury-related gene (*KIM1*) were monitored following treatment with STZ, CM, or EXO by using real-time PCR and the results were presented in Figure 3. The expression of these genes was significantly higher in the DN group than in the control group. However, DN rats treated with CM, EXO, or CM + EXO showed significantly reduced expression, with lowest levels in the co-treated group, compared to rats in the DN group. Again, none of these treatments restored the expression to normal levels. These results indicated that the administration of CM and its EXO could ameliorate fibrosis and renal injury caused by STZ in the rat model of DN with superior effects for the combined therapy.

### 3.5. Effect of CM and/or Its EXOs on Matrix Remodeling-Related Genes

**Figure 4:**
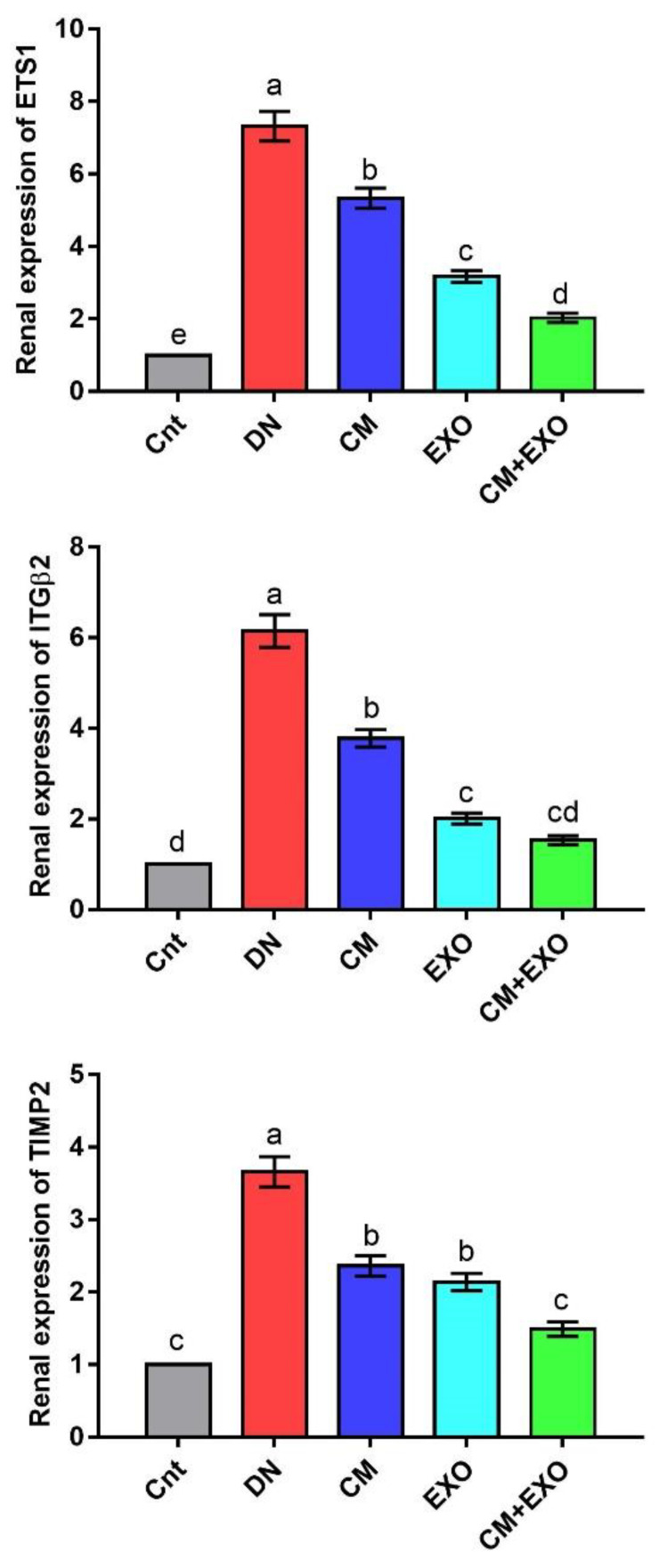
Effect of camel milk and/or its EXOs on renal expression of matrix remodeling genes (*ETS1*, *ITGβ2*, and *TIMP2*) as detected by real-time PCR. Values were expressed as fold change mean ± SEM (n = 6/group). Columns carrying different letters (a (the highest value)–e (the lowest value)) are significantly different at *p* < 0.05. All groups were compared to each other. Each data point represents an average of three independent experiments with three replicates for each. Cnt, control group; DN, diabetic nephropathy group; CM, camel milk-treated group; EXO, camel milk exosomes-treated group; CM + EXO, co-treated group with camel milk and its exosomes.

The renal expression of matrix remodeling-related genes (*ETS1*, *ITGβ2*, and *TIMP2*) was significantly upregulated in the DN group compared to the control group (Figure 4). Individual and combined treatment with CM and its EXOs significantly downregulated *ETS1*, *ITGβ2*, and *TIMP2* expression, with lowest expression in the CM + EXO group, compared to the DN group. Moreover, the expression of *ETS1* and *ITGβ2* was significantly lower in the EXO group than in the CM group. However, no significant difference was noticed in *TIMP2* between the two groups. Interestingly, cotreatment with CM and EXO restored the normal mRNA levels of *ITGβ2* and *TIMP2*. This signified that the administration of CM and its EXO had the potential to inhibit the expression of matrix remodeling genes induced by DN with best improvement when they were given together.

### 3.6. Effects of CM and/or Its EXOs on Renal Histology

**Figure 5:**
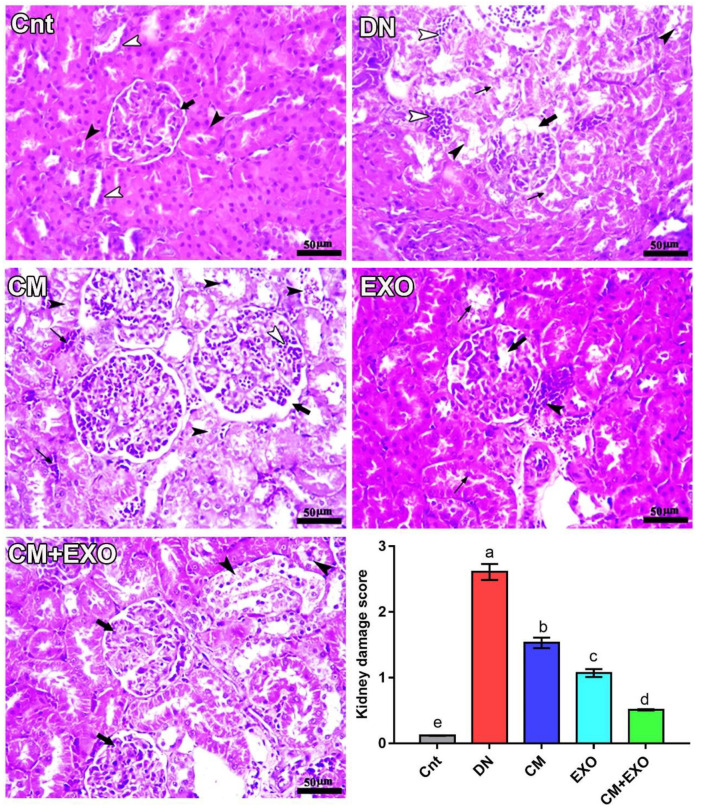
Effect of camel milk and/or its EXOs on renal cortex structure as revealed by histopathological examination of H&E-stained slides, scale bars = 50 µm. All labels (arrows and arrowheads) were explained in the main text. Values of the kidney damage score were expressed as mean ± SEM (n = 6/group). Columns carrying different letters (a (the highest value)–e (the lowest value)) are significantly different at *p* < 0.05. All groups were compared to each other. Cnt, control group; DN, diabetic nephropathy group; CM, camel milk-treated group; EXO, camel milk exosomes-treated group; CM + EXO, co-treated group with camel milk and its exosomes.

Figure 5 shows the histopathological changes in the renal cortex of rats following treatment with STZ, CM, or its EXOs. The control (Cnt) group showed intact renal corpuscle (arrow), containing normal renal glomeruli surrounded by Bowman’s capsule with normal size capsular space in addition to normal proximal (black arrowheads) and distal (white arrowheads) convoluted tubules. In contrast, kidneys of rats in the DN group had notable histopathological alterations including degeneration in renal glomeruli with glomerular sclerosis and mesangial hyperplasia (thick arrow), vacuolar degeneration (thin arrows), and desquamation of tubular epithelium (black arrowheads), in addition to marked inflammatory cells infiltration (white arrowheads). Treatment with CM and its EXOs each alone or in combination (CM + EXO) exhibited slight, moderate, and markable improvement in renal histology, respectively, with the absence of glomerular sclerosis. In the CM group, some renal corpuscles and tubules appeared normal, but others showed some pathological lesions, including mild mesangial hyperplasia (white arrowhead), mild enlargement of capsular space (thick arrow), moderate desquamation and degeneration in renal tubules (black arrowheads), and moderate inflammatory cells infiltration (thin arrows). The EXO group exhibited mild degeneration in some renal glomeruli (thick arrow), mild vacuolar degeneration in some renal tubules (thin arrows), and focal infiltration of inflammatory cells (arrowhead). The CM + EXO group showed notable regenerative changes such as intact renal corpuscles (arrows) but with mild desquamation and degeneration of some renal tubules (arrowheads). As shown in Figure 5, the kidney damage score revealed significantly higher renal damage in the DN group than in the control group. In contrast, the levels of this score were significantly lower in the three treated groups, with the lowest level in the CM + EXO group, rather than in the DN group.

## 4. Discussion

In diabetic patients, chronic hyperglycemia induces a strong inflammatory response, oxidative stress damage, and glomerular hemodynamics which all cause glomerulosclerosis and proteinuria, thereby leading to DN [52]. Because the progress to end-stage renal disease is irreversible, it is necessary to find methods to delay renal damage progress [53]. There are many contradictions regarding the effect of EXOs derived from MSCs, cow, and human milk on diabetes and DN. A previous study reported ameliorated effect [40], but several other studies showed participation in diabetes and DN progression [33,34,35,37]. Unlike bovine and human milk, previous studies have reported beneficial effects of CM on diabetes and attributed this effect to its high levels of insulin [18,19]. Besides, CM-derived EXOs had a proliferative effect on normal pancreatic cells [42]. Therefore, this study aimed to evaluate whether CM and/or its EXOs could ameliorate DN in rats. To the best of our knowledge, this is the first study to report that CM-derived EXOs could attenuate the STZ-induced DN in rats as evidenced by restored kidney function and histology.

DN is characterized by raised urinary excretion of proteins and reduced renal function as indicated by an elevation in creatinine, urea, and RBP serum levels [40,44,52,54]. We also found significantly higher levels of these parameters in DN rats compared to the control group. DN is also associated with a disturbance of redox balance with induction of oxidative markers and inhibition of antioxidant enzyme activities [44,55]. Similarly, in the present study, DN rats exhibited significantly higher renal levels of the lipid peroxidation biomarker (MDA) and significantly lower activities of the antioxidant enzymes (SOD, CAT, and GPx) compared to the control group. Free radical overproduction induces the damage of various cellular components including DNA, proteins, and lipids. To protect these components from oxidative damage, the activities of antioxidant enzymes (such as SOD, GPx, and CAT) increase within the cells to get rid of reactive oxygen species (ROS) [56,57,58]. However, if this antioxidant defense system is interrupted by excessive release of ROS, the cellular components, particularly the cell membrane which is rich in phospholipid, will be damaged by lipid peroxidation [48,59,60].

At a molecular level, many genes such as fibrosis-related genes (*TGFβ1* and *ICAM1*), matrix remodeling-related genes (*ETS1*, *ITGβ2*, and *TIMP2*), and the kidney injury-related gene *KIM1* were involved in the pathogenesis of DN [4,5,6,61]. In support, we also found significantly higher expression of these genes in the renal tissue of DN rats relative to rats in the control group. Elevated free radical levels and the upregulation of *TGFß1* expression are the main hallmarks of DN [55]. Excessive ROS can also induce overexpression of *TGFß1* that exaggerating DN [62]. Upregulated expression of *TGFß1* triggered renal fibrosis through the upregulation of extracellular matrix (ECM)-related genes such as *ICAM1* [61]. Higher expression of *ICAM1* attracted inflammatory cells, particularly macrophages, and T-cells which resulted in the over-release of inflammatory cytokines and chemokines resulting in renal fibrosis [63]. DN patients had higher serum levels of ICAM1 than non-diabetic individuals [64]. TGFß1 also induced the upregulation of the transcription factor *ETS1* [65]. ETS1 plays an important role in the progression of DN through modulation of matrix metalloproteinase expression [66]. Indeed, upregulated expression of *ETS1* is associated with *TIMP2* expression and both genes participated in the progression of DN [5,6]. Moreover, *ETS1* can induce DN in mice through upregulation of its downstream target *ITGβ2* which is involved in matrix remodeling and the activation of neutrophil adherence in DN [6,18].

At the histological level, DN rats showed typical lesions of the nephropathy including degeneration in renal glomeruli with glomerular sclerosis and mesangial hyperplasia. Consistent with our findings, previous studies also reported similar pathological changes in DN animals [6,40,61]. As a result of these deleterious changes, nephrotoxicity caused by diabetes could induce the death of glomerular cells and vasodilatation of glomerular blood vessels [40,67]. Mechanical stretching of podocytes caused by increased blood flow to the kidneys may cause the podocytes foot process to become effaced and the cells to detach causing glomerular filtration abnormalities and proteinuria [68,69]. We also found marked infiltration of inflammatory cells, mainly macrophages. In agreement, Tesch [70] also reported excessive accumulation of macrophages in kidneys of DN rodent models and DN patients, and the degree of infiltration was positively associated with DN progression.

The main findings of the present study were the ameliorative effect of CM and its EXOs against STZ-induced DN as evidenced by restored kidney function (as indicated by the reduction of serum levels of urea, creatinine, and RBP) and redox balance (as revealed by decreasing MDA levels and increasing SOD, CAT, and GPx activities in the kidney), as well as the inhibited expression of *TGFβ1*, *ICAM1*, *ETS1*, *ITGβ2*, *TIMP2*, and *KIM1* and the improved renal histology. A similar ameliorative effect for CM on DN induced by STZ was reported by Korish, Abdel Gader, Korashy, Al-Drees, Alhaider, and Arafah [44] who attributed this effect to CM hypoglycemic, antioxidant and anti-inflammatory properties [10,11,12]. CM is rich in insulin and insulin-like substances which are present within micelles preventing digestion in the stomach [18,19]. CM also had many components that possessed anti-oxidant and anti-inflammatory potential [71], which could also play a crucial role to relieve DN. Whey protein extracted from the CM inhibited the extended inflammation in diabetic rats by decreasing the release of pro-inflammatory cytokines such as TNFα, IL6, and IL1β [72]. CM also downregulated the expression of TNFα, IL1β, and NFκB in the kidney of 5-fluorouracil-treated rats [73].

To the best of our knowledge, this is the first study to report that CM-derived EXOs could attenuate the STZ-induced DN in rats. In agreement, EXOs derived from BM-MSCs also relieved the deleterious effects induced by STZ in the rat model of DN and improved renal function and structure [40]. Moreover, CM and MSCs-derived EXOs induced the proliferation of normal human pancreatic H6c7 cells [42] and pancreatic β-cells [41], respectively. As most EXO actions are mediated through their cargo of miRNAs, the effect of CM-derived EXOs reported in our study could be attributed to the miRNAs-mediated paracrine effect of these EXOs. Exosomal miRNAs could inhibit the expression of inflammation, fibrosis, and matrix remodeling genes and control the expression of antioxidant genes. Indeed, previous studies have reported antioxidant effects for CM-EXOs against oxidative effects induced by cyclophosphamide and breast cancer in rats [32,45]. Additionally, CM and its EXOs inhibited *IL6*, *TNFα*, and *NFκB* expression in spleens of immunocompromised rats and mammary tumor tissues [32,45]. In contrast, several other studies reported stimulatory effects for EXOs derived from cow/human milk, pancreatic β-cells or stem cells during DN pathogenesis. A previous study reported that EXOs derived from rats and human pancreatic β-cells participated effectively in the pathogenesis of type I diabetes through the activation of dendritic cells, which induced cytokine release and apoptosis of β-cells [33]. EXOs derived from MSCs resident in the pancreas of diabetic mice induce T cell-mediated destruction of β-cells [34]. Bone marrow (BM) MSC-derived EXOs loaded with miR-let7a-5p could induce DN [35]. Moreover, urine stem cell-derived EXOs loaded with miR-145 and miR-320c induced renal fibrosis through activation of TGFβ1 expression [36]. BM-MSCs-EXOs can also induce glomerular sclerosis by activating the PI3K/Akt/mTOR pathway [37]. Adipose tissue MSC-derived EXOs loaded with miR-486 triggered the overproduction of free radicals that modulate the PI3K/Akt/mTOR pathway leading to apoptosis of podocytes and DN progression [38]. The contradictory results regarding the effect of EXOs on DN may be attributed to the source of EXOs which could produce different exosomal miRNAs. Therefore, further studies are required to investigate the miRNAs content of camel milk EXOs and compare them with those derived from cow and human milk as well as MSCs. Further investigations are also required to perform proteomics, metabolomics, and RNA-seq of the exosomes from milk of different animals such as camel, goat, cow, and buffalo to compare their cargoes. As a bad side of EXOs, cancer cell-derived EXOs play a crucial role in the formation and progression of tumor. Recent studies used advanced tools such as localized surface plasmon resonance (LSPR), atomic force microscopy (AFM), and self-assembly gold nanoislands (SAM-AuNIs) to effectively detect these exosomal biomarkers [74,75,76,77,78,79,80]. It would be worth applying this advanced biosensing technology to biosense animal’s health via milk-derived, exosome-based biomarkers.

## 5. Conclusions

Camel milk and its exosomes ameliorated the deleterious effects associated with STZ-induced diabetic nephropathy in rats through reduction of kidney damage scores, maintenance of redox balance, and inhibition of fibrosis and matrix remodeling-related genes. Thus, camel milk exosomes can be used as novel agents for the treatment strategies of diabetic nephropathy in the future. Further investigations are required to explain the exact molecular mechanisms underlying their effects with a special focus on miRNAs, proteins, and metabolites cargoes of exosomes.

## Figures and Tables

**Table 1:** Primers used for real-time PCR.

Gene	Forward Primer (5′-----3′)	Reverse Primer (5′-----3′)
*TGFβ1*	ACGTCAGACATTCGGGAAGCAGTG	GCAAGGACCTTGCTGTACTGTGTG
*ICAM1*	GCAACCCCATCAAGAGGATTC	GGGGCCGTGTAGATAAACTCG
*KIM1*	TGGCACTGTGACATCCTCAGA	GCAACGGACATGCCAACATA
*ETS1*	CCTGCAGATTGTTCCGGAGT	CTGGGGCCAC CTTTACTGAG
*ITGβ2*	GTTTCAGACAGAGGTCGGCA	AATTTCCTCCGGACAGGCAG
*TIMP2*	GCAACCCCATCAAGAGGATTC	GGGGCCGTGTAGATAAACTCG
*β-actin*	AAGTCCCTCACCCTCCCAAAAG	AAGCAATGCTGTCACCTTCCC

**Table 2:** Effect of camel milk and/or its EXOs on levels of renal damage parameters and fasting blood glucose.

Groups	Urea (mg/dL)	Creatinine (mg/dL)	Retinol Binding Protein (ng/mL)	Urinary Proteins (mg/mL)	Fasting Blood Glucose (mg/dL)
Cnt	16.11 ± 0.85 ^e^	0.77 ± 0.04 ^d^	36.22 ± 1.49 ^e^	0.32 ± 0.01 ^d^	109.38 ± 5.66 ^d^
DN	72.32 ± 3.58 ^a^	2.10 ± 0.10 ^a^	215.54 ± 10.15 ^a^	1.90 ± 0.08 ^a^	348.60 ± 17.90 ^a^
CM	56.49 ± 2.17 ^b^	1.55 ± 0.08 ^b^	159.06 ± 7.42 ^b^	1.52 ± 0.06 ^b^	236.09 ± 12.00 ^b^
EXO	42.85 ± 2.03 ^c^	1.39 ± 0.07 ^b^	119.37 ± 5.38 ^c^	1.43 ± 0.06 ^b^	219.27 ± 11.62 ^b^
CM + EXO	34.27 ± 1.42 ^d^	1.04 ± 0.06 ^c^	88.73 ± 4.40 ^d^	0.95 ± 0.03 ^c^	177.46 ± 8.11 ^c^

Data are presented as mean ± SEM (n = 6/group). Columns carrying different letters (a (the highest value)–e (the lowest value)) are significantly different at *p* < 0.05. All groups were compared to each other. Each data point represents an average of three independent experiments with three replicates for each. Cnt, control group; DN, diabetic nephropathy group; CM, camel milk-treated group; EXO, camel milk exosomes-treated group; CM + EXO, co-treated group with camel milk and its exosomes.

## Data Availability

The data supporting the present findings are contained within the manuscript.
